# Integral Velocidade-Tempo da Insuficiência Aórtica: Um Novo Marcador Ecocardiográfico na Avaliação da Gravidade da Insuficiência Aórtica

**DOI:** 10.36660/abc.20190243

**Published:** 2020-08-19

**Authors:** José Abellán-Huerta, Juan Carlos Bonaque-González, Ramón Rubio-Patón, José García-Gómez, Santiago Egea-Beneyto, Federico Soria-Arcos, Luciano Consuegra-Sánchez, Rosa María Soto-Ruiz, José Luis Ramos-Martín, Juan Antonio Castillo-Moreno

**Affiliations:** 1 Departamento de Cardiologia Hospital General Universitario Santa Lucía Cartagena Spain Departamento de Cardiologia, Hospital General Universitario Santa Lucía, Cartagena, Spain

**Keywords:** Insuficiência Cardíaca, Insuficiência da Valva Aórtica/diagnóstico por imagem, Ecocardiografia, Doppler/métodos

## Abstract

**Fundamento:**

A ecocardiografia é essencial para o diagnóstico e a quantificação da insuficiência aórtica (IA). A integral velocidade-tempo (IVT) do fluxo da IA pode estar relacionada à gravidade da IA.

**Objetivo:**

Este estudo tem por objetivo avaliar se a IVT é um marcador ecocardiográfico de gravidade da IA.

**Métodos:**

Foram incluídos todos os pacientes com IA nativa moderada ou grave e ritmo sinusal que visitaram o nosso laboratório de imagem entre janeiro e outubro de 2016. Todos os indivíduos foram submetidos a um ecocardiograma completo com medição da IVT da IA. A associação entre a IVT e a gravidade da IA foi analisada por regressão logística e modelos de regressão multivariada. Valores p<0,05 foram considerados estatisticamente significativos.

**Resultados:**

Entre os 62 pacientes incluídos (68,5±14,9 anos; 64,5%: IA moderada; 35,5%: IA grave), a IVT foi maior em indivíduos com IA moderada em comparação àqueles com IA grave (2,2±0,5 m versus 1,9±0,5 m, p=0,01). Pacientes com IA grave apresentaram valores maiores de diâmetro diastólico final do ventrículo esquerdo (DDFVE) (56,1±7,1 mm versus 47,3±9,6 mm, p=0,001), volume diastólico final do ventrículo esquerdo (VDFVE) (171±36,5 mL versus 106±46,6 mL, p<0,001), orifício regurgitante efetivo (0,44±0,1 cm2 versus 0,18±0,1 cm2, p=0,002) e volume regurgitante (71,3±25,7 mL versus 42,5±10,9 mL, p=0,05), assim como menor fração de ejeção do ventrículo esquerdo (FEVE) (54,1±11,2% versus 63,2±13,3%, p=0,012). A IVT mostrou ser um marcador de gravidade da IA, independentemente do DDFVE, VDFVE e FEVE ( *odds ratio* 0,160, p=0,032) e da frequência cardíaca e pressão arterial diastólica (PAD) ( *odds ratio* 0,232, p=0,044).

**Conclusões:**

A IVT do fluxo da IA apresentou associação inversa com a gravidade da IA, independentemente do diâmetro e volume do ventrículo esquerdo, frequência cardíaca, PAD e FEVE. A IVT pode ser um marcador de gravidade da IA em pacientes com IA nativa e ritmo sinusal. (Arq Bras Cardiol. 2020; [online].ahead print, PP.0-0)

## Introdução

Insuficiência aórtica (IA) é uma das incompetências valvulares mais comuns no mundo desenvolvido.^1^ O controle típico da doença envolve uma combinação de sinais e sintomas clínicos e coleta de dados por meio de exames complementares. A ecocardiografia é uma ferramenta fundamental para o diagnóstico e a quantificação da IA^2^ e sua interpretação correta requer uma abordagem que integra medidas e parâmetros qualitativos, semiquantitativos e quantitativos.^3 , 4^ No entanto, estes parâmetros não estão isentos de limitações.^3^

A integral velocidade-tempo (IVT) é definida como a área medida abaixo da curva de velocidade Doppler em um determinado ponto. No caso da IA, este valor corresponde ao gradiente de pressão diastólica entre a aorta e o ventrículo esquerdo (VE).^5^ Em pacientes com IA, a IVT é multiplicada pelo orifício regurgitante efetivo (ORE) da aorta para calcular o volume regurgitante (VR) (VR=ORExIVT).^2 , 6 , 7^ Este parâmetro tem demonstrado sua eficácia na determinação da gravidade da IA, mesmo com o valor do ORE sendo calculado com base no método de área da superfície de isovelocidade proximal ( *proximal isovelocity surface area* – PISA), que é conhecido por ter limitações inerentes em pacientes com IA.^3 , 8 , 9^ Além disso, tendo em conta a referida equação, pacientes com IA grave geralmente apresentam valores maiores de VR^2^ e ORE,^7 , 10^ mas não há evidência do comportamento da IVT em relação à gravidade da IA.

Ademais, pacientes com IA grave costumam ter pressão diastólica final do VE elevada,^11^ bem como pressão arterial diastólica (PAD) reduzida.^12 , 13^ Estas mudanças de pressão podem diminuir a IVT fisiopatologicamente por meio da redução do gradiente de pressão entre a aorta e o VE. Este estudo tem por objetivo determinar se a IVT é um marcador ecocardiográfico de gravidade da IA.

## Métodos

### Delineamento e População do Estudo

Este estudo observacional transversal retrospectivo foi realizado durante dez meses (de janeiro a outubro de 2016). Todos os pacientes com IA que visitaram o nosso laboratório de imagem cardíaca durante este período foram considerados elegíveis. Os pacientes tinham que apresentar IA moderada a grave em uma valva nativa (valva não-protética), além de assinar um termo de consentimento livre e esclarecido para serem incluídos no estudo. Foram excluídos os pacientes com fibrilação atrial ou evidência de qualquer tipo de arritmia, jatos múltiplos ou excêntricos de IA. O estudo foi conduzido em conformidade com a declaração de Helsinki e foi aprovado pelo comitê de ética da nossa comissão de pesquisa local.

### Características de Referência da População

Todos os participantes do estudo tiveram as seguintes informações demográficas e clínicas coletadas: idade, sexo, histórico de hipertensão arterial, dislipidemia, diabetes mellitus e tabagismo. Qualquer tipo de medicamento anti-hipertensivo, hipolipemiante ou antiarrítmico que os participantes estivessem tomando no momento da inclusão no estudo também foram registrados. Durante o ecocardiograma, a altura e o peso de cada paciente foram coletados e três aferições da pressão arterial foram realizadas após 5 minutos de repouso, usando um monitor de pressão arterial M6 Comfort HEM-7221-E8 (Omron Healthcare, Kioto, Japão) – validado por protocolos da Dabl®Educational Trust e da Sociedade Britânica de Hipertensão –, de acordo com as recomendações da Sociedade Europeia de Hipertensão/Sociedade Europeia de Cardiologia ( *European Society of Hypertension/European Society of Cardiology* – ESH/ESC).^14^ A pressão arterial final foi a média da segunda e terceira aferições. A frequência cardíaca (FC) foi determinada no momento da medição da IVT da IA. Todos os pacientes também tiveram um exame de sangue realizado imediatamente após a coleta para determinar o nível de creatinina plasmática e calcular a taxa de filtração glomerular, com base na equação CKD-EPI ( *Chronic Kidney Disease – Epidemiology Collaboration* ).^15^ O analisador hematológico usado foi o PE Chemistry (Roche Diagnósticos, Manheim, Alemanha).

### Variáveis Ecocardiográficas

**Figura 1:**
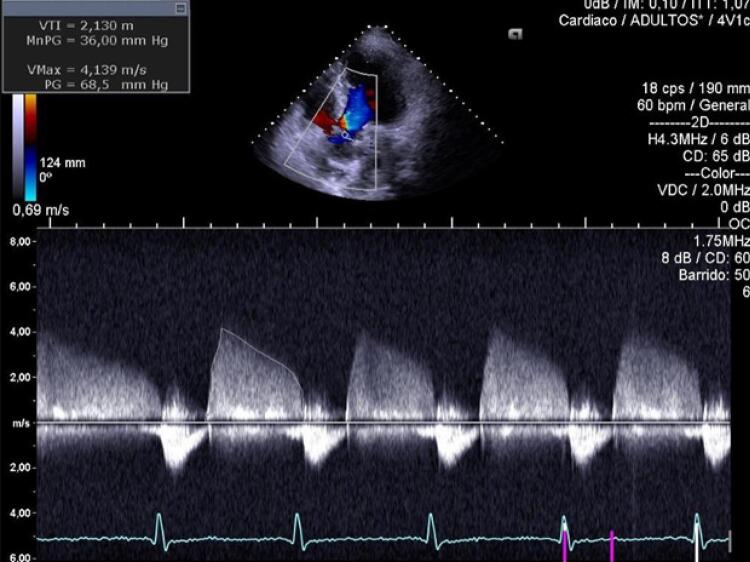
**–** Medida da integral velocidade-tempo do fluxo da insuficiência aórtica a partir do corte apical 5 câmaras. MnPG: gradiente médio de pressão; PG: gradiente máximo de pressão; Vmax: velocidade máxima do fluxo da insuficiência aórtica; VTI: integral velocidade-tempo da insuficiência aórtica.

Ecocardiogramas foram realizados em todos os participantes, utilizando o sistema de ultrassonografia Acuson Siemens SC2000. O método de Simpson biplano foi empregado para obter medições, imagens e vídeos padrão, incluindo o diâmetro diastólico final do ventrículo esquerdo (DDFVE), diâmetro sistólico final do ventrículo esquerdo (DSFVE) e a fração de ejeção do ventrículo esquerdo (FEVE), em conformidade com as recomendações da Sociedade Americana de Ecocardiografia.^16^ Tanto a espessura quanto o diâmetro foram determinados em modo M com um alinhamento adequado sempre que possível; caso contrário, as medições foram feitas em 2D. A medição da IVT do fluxo da IA foi realizada com registros de Doppler contínuo a partir do corte que mostrasse o melhor alinhamento com o jato regurgitante, em particular, o corte apical 5 câmaras ( Figura 1 ) ou o paraesternal eixo longo em casos de jato regurgitante vertical. Considerando que a FC se comporta como um determinante temporal da IVT da aorta, além da IVT absoluta, o índice IVT (iIVT) também foi calculado por meio da divisão da IVT pela FC (iIVT=IVT/FC). A morfologia das valvas aórticas foi examinada a partir do corte paraesternal eixo curto. Os diâmetros sistólicos da via de saída dos ventrículos direito e esquerdo também foram medidos. O tempo de meia-pressão (TMP) foi calculado utilizando o corte apical 5 câmaras. A vena contracta (VC) foi estimada com Doppler colorido em dois planos ortogonais, de acordo com as recomendações.^16^ O ORE foi calculado por meio do método PISA.^10 , 17^ Para isso, imagens do fluxo regurgitante foram obtidas usando o melhor corte possível para o alinhamento do fluxo convergente. Ao ampliar este corte, a escala do Doppler colorido foi otimizada até o que hemisfério de isovelocidade pudesse ser devidamente diferenciado. O raio PISA foi medido entre a primeira circunferência de *aliasing* em relação ao centro do hemisfério em protodiástole, no momento exato em que o fluxo regurgitante atinge a velocidade máxima. O VR foi definido como o produto de ORExIVT. Ademais, sempre que possível, o VR também foi determinado quantitativamente pela estimativa do volume sistólico aórtico e pulmonar.^18^ O fluxo reverso na aorta torácica foi estabelecido por Doppler pulsado na extremidade proximal da aorta descendente por meio do corte supraesternal. O fluxo holodiastólico com velocidade diastólica final >20 cm/s foi considerado como fluxo reverso positivo. Por fim, após uma análise abrangente e integrativa dos diferentes registros estruturais e qualitativos do Doppler e dos parâmetros semiquantitativos obtidos e considerando as recomendações mais recentes,^3 , 6^ dois ecocardiografistas experientes quantificaram a IA separadamente. Um terceiro ecocardiografista experiente avaliou e quantificou a IA de forma conclusiva, em caso de discordância entre os dois primeiros cardiologistas.

### Análise Estatística

A distribuição normal foi testada em todas as variáveis pelo teste de Kolmogorov-Smirnov. Variáveis contínuas com distribuição normal foram expressas como média±desvio padrão (DP) e as com distribuição assimétrica como mediana [intervalo interquartil (IIQ)]. As variáveis categóricas foram expressas como porcentagem. As correlações foram estudadas por meio do método de Spearman ou de Pearson, conforme apropriado. A variabilidade inter-observador para a quantificação da gravidade da IA foi determinada pelo coeficiente de correlação intraclasse e plotagens de Bland-Altman.^19^ Análises de confiabilidade utilizando a estatística kappa (κ) definiram a conc^o^rdância entre os dois ecocardiografistas quanto à gravidade da IA (moderada ou grave). Diferenças iniciais entre pacientes com IA moderada ou grave foram avaliadas pelo teste t de Student não pareado ou pelo teste U de Mann-Whitney para as variáveis contínuas e pelo teste χ^2^ para as variáveis categóricas. A análise de regressão logística avaliou a associação entre cada variável de referência e a IA grave. Modelos de regressão logística multivariada determinaram as variáveis independentemente associadas com IA grave. As variáveis incluídas foram aquelas com p<0,05 na análise univariada, excluindo o VR, o ORE e a VC por não estarem disponíveis para todos os pacientes, o que poderia causar sobreajuste. O desempenho do modelo em prever a IA grave foi avaliado por medidas de calibração (estatística de Hosmer-Lemeshow) e discriminação (índice C), ambas internamente validadas utilizando a técnica de reamostragem *bootstrap* . A associação entre a IVT da IA e sua gravidade foi explorada por meio de análise multivariada, independentemente da FC e da PAD. A relação entre o iIVT e a gravidade da IA foi avaliada em uma nova análise de regressão logística. Intervalos de confiança (IC95%) foram fornecidos quando necessário. Todos os valores de probabilidade foram bilaterais e valores p<0,05 foram considerados estatisticamente significativos. A análise estatística foi realizada utilizando o programa SPSS, v.18.0 (SPSS Inc., Chicago, Ilinois).

## Resultados

A amostra original foi composta de 65 pacientes com IA nativa moderada ou grave em ritmo sinusal. Não foi possível obter um alinhamento adequado do Doppler para o jato regurgitante em três pacientes, que apresentaram jatos muito excêntricos, sendo portanto excluídos. Dos 62 participantes restantes, 40 (64,5%) tinham IA moderada e 22 (35,5%), IA grave. IA aguda foi diagnosticada em 4 pacientes (6,5% da amostra). A concordância entre a quantificação definida pelos dois ecocardiografistas foi κ=0,83. Todos os pacientes incluídos eram caucasianos. As características de referência da amostra estão presentes na Tabela 1 .

**Tabela 1:** – Características de referência

Características	Total (n=62)	IA moderada (n=40)	IA grave (n=22)	Valor p
**Idade (anos)**	68,5±14,9	68,6±14,2	66,1±15,5	0,299
**Masculino**	33 (53,2)	20 (50)	13 (59,1)	0,492
**IMC (kg/m** ^**2**^ **)**	27,5±4,7	26,5±4	29,4±5,9	0,340
**PAS (mmHg)**	135,6±17,8	133,6±16,7	139,4±19,8	0,213
**PAD (mmHg)**	62,2±15,5	63,2±12,7	59,8±19,8	0,373
**Frequência cardíaca**	66,8±11,3	65,8±10,6	68,5±12,4	0,382
**Hipertensão arterial**	45 (72,6)	29 (72,5)	16 (72,7)	0,985
**Diabetes mellitus**	11 (17,7)	8 (20)	3 (16,6)	0,530
**Dislipidemia**	30 (48,4)	20 (50)	10 (45,5)	0,732
**Fumantes ativos**	10 (16,1)	6 (15)	4 (18,2)	0,744
**TFGe (mL/min/1,73 m** ^**2**^ **)**	77,3 [40,3]	86,6 [42,3]	72,9 [34,6]	0,408
**Hemoglobina**	13,2±1,8	13,3±1,8	13,2±2	0,893
**Betabloqueadores**	29 (46,8)	17 (42,5)	12 (54,5)	0,363
**Inibidores da ECA**	19 (30,6)	11 (27,5)	8 (36,4)	0,469
**ARA**	16 (25,8)	13 (32,5)	3 (13,6)	0,104
**BCC DHP**	2 (3,2)	2 (5)	0 (0)	0,286
**BCC não-DHP**	10 (16,1)	4 (10)	6 (27,3)	0,145
**Amiodarona**	2 (3,2)	1 (2,5)	1 (4,5)	1
**Diuréticos**	31 (50)	17 (42,5)	14 (63,6)	0,111
**Estatina**	26 (41,9)	19 (47,5)	7 (31,8)	0,231
**Hospitalização prévia por IC**	16 (25,8)	9 (22,5)	7 (31,8)	0,422

*ECA: enzima conversora de angiotensina; IA: insuficiência aórtica; ARA: antagonistas do receptor de angiotensina; IMC: índice de massa corporal; BCC: bloqueadores dos canais de cálcio; PAD: pressão arterial diastólica; DHP: dihidropiridina; TFGe: taxa de filtração glomerular estimada; IC: insuficiência cardíaca; PAS: pressão arterial sistólica. Variáveis contínuas com distribuição normal são expressas como média±desvio padrão, as com distribuição assimétrica como mediana [intervalo interquartil] e as variáveis categóricas como n (porcentagem).*

Como mostra a Tabela 2 , a IVT do fluxo regurgitante da aorta foi maior em pacientes com IA moderada em relação àqueles com IA grave. O intervalo da IVT foi 2,05 m (1,53–3,58 m) no grupo com IA moderada e 1,88 m (0,96–2,84 m) no grupo com IA grave. Uma correlação inversa e significativa foi identificada entre a IVT e a FC [coeficiente de correlação de Pearson (r_p_)=-0,408, p=0,001]. Pacientes com IA grave apresentaram menor FEVE e maior DDFVE, DSFVE, ORE, VR e VC. No entanto, a medida correta destes parâmetros só foi possível em 62,9% da amostra para o ORE, 67,7% para o VR e 72,6% para a VC. Salienta-se que não foi identificada associação estatisticamente significativa entre a gravidade da IA e o TMP, embora uma tendência para isso tenha sido detectada.

**Tabela 2:** – Valores de parâmetros ecocardiográficos

Parâmetro	Total (n=62)	IA moderada (n=40)	IA grave (n=22)	Valor p
**IVT IA (m)**	2,1±0,5	2,2±0,5	1,9±0,5	0,010
**iIVT IA (IVT/frequência cardíaca)**	0,033±0,012	0,036±0,013	0,028±0,01	0,024
**TMP aórtica (ms)**	397,3±110,1	434,2±127	367,5±86,2	0,062
**Vena contracta (mm)**	6±1,5	5,5±1,5	7,1±1,2	0,035
**ORE (cm** ^**2**^ **)**	0,31±0,2	0,18±0,1	0,44±0,1	0,002
**Volume regurgitante (mL)**	56,9±24	42,5±10,9	71,3±25,7	0,05
**Fluxo reverso na aorta torácica**	33 (53,2)	12 (30,8)	21 (95,5)	<0,001
**Espessura do septo IV (mm)**	13,1±3,6	12,5±3,5	13,8±3,5	0,460
**Espessura da parede posterior (mm)**	10,6±2,8	10,3±2,7	11±3,1	0,383
**DDFVE (mm)**	50,5±9	47,3±9,6	56,1±7,1	0,001
**DSFVE (mm)**	31±11,4	26,9±12,3	38,4±8,1	<0,001
**VDFVE (mL)**	131,9±54,3	106±46,6	171±36,5	<0,001
**VSFVE (mL)**	53,6±36,1	39,9±32,2	78,7±27,5	<0,001
**FEVE (%)**	59,7±13,2	63,2±13,3	54,1±11,2	0,012
**Velocidade de pico da IA (m/s)**	4,2±0,51	4,3±0,5	4,1±0,52	0,344
**Velocidade de pico sistólico da aorta (m/s)**	2,7±1,2	2,8±1,4	2,7±0,9	0,791
**Valva aórtica bicúspide**	5 (8,1)	3 (4,8)	2 (3,2)	0,826
**Pressão de enchimento do VE elevada**	26 (41,9)	16 (42,1)	10 (50)	0,566
**Insuficiência mitral grave**	2 (3,2)	2 (5)	0 (0)	0,286
**Estenose mitral grave**	1 (1,6)	1 (2,5)	0 (0)	0,455
**Estenose aórtica grave**	8 (12,9)	6 (15)	2 (9,1)	0,507

*IA: insuficiência aórtica; ORE: orifício regurgitante efetivo; IV: interventricular: VE: ventrículo esquerdo; FEVE: fração de ejeção do ventrículo esquerdo; DDFVE: diâmetro diastólico final do ventrículo esquerdo; VDFVE: volume diastólico final do ventrículo esquerdo; DSFVE: diâmetro sistólico final do ventrículo esquerdo; VSFVE: volume sistólico final do ventrículo esquerdo; TMP: tempo de meia-pressão; IVT: integral velocidade-tempo; iIVT: índice integral velocidade-tempo. Variáveis contínuas com distribuição normal são expressas como média±desvio padrão e as variáveis categóricas como n (porcentagem).*

Na análise bivariada ( Tabela 3 ), a IVT foi inversamente associada com a gravidade da IA. Além disso, as variáveis clássicas de gravidade relacionadas ao tamanho e à função do ventrículo esquerdo foram associadas com a gravidade da IA. Na análise multivariada, o valor da IVT funcionou como um marcador de gravidade da IA, independentemente do DDFVE, do volume diastólico final do ventrículo esquerdo (VDFVE) e da FEVE ( Tabela 4 ). O DSFVE e o volume sistólico final do ventrículo esquerdo foram excluídos da análise multivariada pela colinearidade com o DDFVE (r_p_=0,905, p<0,001) e o VDFVE (r_p_=0,871, p<0,001), respectivamente. Também foram excluídos da análise multivariada o ORE, o VR e a VC, já que eles não puderam ser obtidos para todos os pacientes devido à janela ultrassonográfica inadequada ou dificuldade na medição. Este modelo mostrou maior discriminação (estatística C=0,837, IC95% 0,728–0,947) e uma calibração precisa (Hosmer-Lemeshow χ^2^=2,30, p=0,970).

**Tabela 3:** – Modelo de regressão logística bivariada (variável dependente: insuficiência aórtica grave)

	*Odds ratio*	IC95%	Valor p
IVT IA	0,198	0,053–0,748	0,017
iIVT IA	<0,001	<0,001–0,005	0,033
FEVE	0,941	0,895–0,989	0,017
DDFVE	1,144	1,047–1,249	0,003
DSFVE	1,119	1,044–1,199	0,001
VDFVE	1,032	1,015–1,049	<0,001
VSFVE	1,034	1,013–1,057	0,002

*IC95%: intervalo de confiança de 95%; IVT IA: integral velocidade-tempo da insuficiência aórtica; iIVT IA: índice integral velocidade-tempo da insuficiência aórtica; DDFVE: diâmetro diastólico final do ventrículo esquerdo; VDFVE: volume diastólico final do ventrículo esquerdo; FEVE: fração de ejeção do ventrículo esquerdo; DSFVE: diâmetro sistólico final do ventrículo esquerdo; VSFVE: volume sistólico final do ventrículo esquerdo.*

**Tabela 4:** – Modelo de regressão logística multivariada (variável dependente: insuficiência aórtica grave)

	*Odds ratio*	IC95%	Valor p
IVT IA	0,160	0,030–0,856	0,032
FEVE	1,005	0,933–1,082	0,895
DDFVE	1,049	0,934–1,178	0,419
VDFVE	1,030	1,009–1,052	0,005

	***Odds ratio***	**IC95%**	**Valor p**

iIVT IA	<0,001	<0,001–<0,001	0,019
FEVE	1,007	0,932–1,089	0,859
DDFVE	1,063	0,939–1,204	0,333
VDFVE	1,032	1,010–1,055	0,005

*IC95%: intervalo de confiança de 95%; IVT IA: integral velocidade-tempo da insuficiência aórtica; iIVT IA: índice integral velocidade-tempo da insuficiência aórtica; DDFVE: diâmetro diastólico final do ventrículo esquerdo; VDFVE: volume diastólico final do ventrículo esquerdo; FEVE: fração de ejeção do ventrículo esquerdo.*

Por outro lado, como a FC e a PAD poderiam influenciar fisiopatologicamente a medição da IVT (PAD como determinante de velocidade e FC, de tempo), a associação entre a IVT e a gravidade da IA foi avaliada com ajuste para FC e PAD. A IVT também foi inversamente relacionada à gravidade da IA, independentemente desses fatores (OR 0,232, IC95% 0,056–0,961, p=0,044). Por último, o iIVT da IA também mostrou associação inversa com a gravidade da IA ( Tabela 3 ) e agiu como um marcador de gravidade da IA, independentemente do DDFVE, VDFVE e FEVE na análise multivariada ( Tabela 4 ). Além disso, esta variável também foi relacionada à gravidade da IA, independentemente da PAD (OR<0,001, IC95% <0,001–0,001, p=0,029).

## Discussão

Este estudo sugere que a IVT da IA pode ser usada como um marcador de gravidade em pacientes com IA significativa, considerando que a estimativa da gravidade por meio da ecocardiografia é um processo difícil que envolve a integração de vários exames e parâmetros diferentes.^2 - 4 , 20^

Efetivamente, o ORE pelo método PISA funciona como um parâmetro de estratificação da gravidade da IA^3 , 7^ e uma relação indireta pode ser identificada entre o ORE e a IVT (ORE=VR/IVT).^3 , 10^ A IA mais grave apresenta ORE e VR maiores, mas o comportamento da IVT é desconhecido. Neste estudo, a IVT do fluxo regurgitante da aorta foi inversamente associada com a gravidade da IA. Há pouca evidência científica disponível corroborando essa relação. Zarauza et al.,^21^ publicaram um estudo que avaliou o valor da IVT da IA, entre outros parâmetros, em uma amostra de 43 pacientes com IA moderada a grave.^21^ Os autores revelaram achados semelhantes aos encontrados na presente investigação (IVT da IA grave: 1,8±0,7 m versus 1,9±0,5 m; IVT da IA moderada: 2,2±0,8 m versus 2,2±0,5 m, respectivamente). Contudo, no estudo de Zarauza et al.,21 as diferenças entre a IVT da IA grave e moderada não alcançaram significância estatística. A diferença no tamanho da amostra de pacientes com IA moderada (15 versus 40) poderia explicar a falta de um resultado significativo. Até onde sabemos, nenhum estudo avaliou o valor da IVT como um indicador de gravidade da IA.

Um aspecto notável do presente estudo é a associação direta entre a gravidade da IA e os diâmetros e volumes diastólico final e sistólico final, além da relação inversa com a FEVE. Estes resultados corroboram as evidências científicas disponíveis, que defendem o papel preditivo do diâmetro do ventrículo esquerdo e da função ventricular como marcadores de IA avançada e prognósticos negativos.^11 , 22 - 24^ Acredita-se que este aspecto reflete uma metodologia adequada e rigorosa para a medição desses parâmetros. Este estudo identificou que a relação entre a IVT e a gravidade da IA não depende de variáveis ecocardiográficas, como diâmetros, volumes ou fração de ejeção do ventrículo esquerdo. Este resultado poderia potencialmente respaldar o uso da IVT como indicador na maioria dos cenários ecocardiográfico envolvendo a IA e o ritmo sinusal.

Apesar de serem métodos ecocardiográficos recomendados para determinar a gravidade da IA significativa,^3 , 25 , 26^ os cálculos necessários para estimar a VC, o VR e, como já mencionado anteriormente, o ORE obtido pelo método PISA apresentam diversas limitações.^3 , 8 , 9 , 17^ De fato, este estudo não pôde avaliar se o valor da IVT estava associado com a IA grave, independentemente do ORE, VR ou VC, pois a porcentagem de pacientes para os quais estes dados puderam ser obtidos não foi suficiente para realizar uma análise multivariada válida. Em contraste, a IVT não pôde ser estimada em apenas 3 dos 65 pacientes deste estudo devido a um alinhamento inadequado do jato da IA. Assim, a IVT mostrou ser um parâmetro reprodutível, que pode ser facilmente obtido e examinado na maioria dos pacientes, sendo capaz de fornecer informações valiosas para a estratificação da gravidade da IA.

Destaca-se também que, apesar do TMP ter sido obtido para todos os pacientes que tiveram a IVT calculada, não foram encontradas diferenças significativas entre indivíduos com IA moderada e grave, o que impossibilitou a inclusão deste parâmetro na análise multivariada. Portanto, não foi possível avaliar o valor adicional da IVT com relação ao TMP. Diretrizes clínicas atuais sugerem que o aproveitamento do TMP é baixo em casos de IA crônica^2 , 3^ e a amostra do presente trabalho é composta sobretudo de pacientes com IA crônica. A baixa taxa de IA aguda neste estudo (6,5%) impediu uma avaliação estatística viável de coorte da IA aguda. Esta situação poderia explicar a ausência de diferenças entre os valores do TMP dos grupos com IA moderada e grave.

Os resultados deste estudo também sugerem que a associação entre a IVT baixa e a IA grave não parece ser significativamente afetada por variáveis hemodinâmicas, tais como a FC e a PAD. Se outros estudos fundamentarem este comportamento de relacionamento, o uso da IVT poderá alcançar uma grande variedade de pacientes. No entanto, considera-se que a relação entre a IVT e a gravidade da IA não seria significativamente alterada por estas variáveis hemodinâmicas devido à falta de valores extremos. Ressalta-se que uma tendência de PAD mais baixa foi identificada em pacientes com IA grave e que pacientes com fibrilação atrial foram excluídos. Assim, considerando que a FC é um determinante temporal para a IVT da IA, a IVT indexada por FC também foi calculada para normalizar o valor da IVT e aprofundar o estudo da sua relação com a gravidade da IA. Além disso, a FC foi correlacionada inversa e significativamente com a IVT da IA. A relação entre esta nova variável e a gravidade da IA foi não apenas mantida, mas se mostrou mais forte e independente do DDFVE e da FEVE (OR<0,001, p=0,031). Em outros estudos, como o de Zarauza et al.,^21^ a IVT foi normalizada utilizando o comprimento diastólico.^21^ Todavia, existem poucos níveis de consistência na indexação da IVT em termos de FC. Acredita-se que estes achados reforçam a hipótese fisiopatológica de que uma IVT menor esteja associada a uma IA mais grave, independentemente da FC.

Este estudo apresenta diversas limitações. Primeiro, este é um estudo unicêntrico que não analisou pacientes com IA e fibrilação atrial ou prótese valvar; portanto, o valor da IVT aórtica nestas subpopulações é desconhecido. Segundo, a IVT foi obtida por Doppler e, consequentemente, está sujeita às limitações desta técnica. Ademais, as análises incluíram apenas pacientes com IA moderada ou grave, em uma tentativa de evitar uma possível subestimação da medição da IVT de jatos regurgitantes leves de baixa densidade; assim, a eficácia da IVT em determinar a gravidade da IA leve permanece incerta. ORE, VR e VC não puderam ser obtidos para todos os pacientes, em parte pela natureza retrospectiva do presente trabalho, impedindo a avaliação do valor da IVT em relação a estes parâmetros na previsão de IA grave em uma análise multivariada. Nenhuma outra técnica exploratória, como ecocardiograma transesofágico, ultrassonografia 3D ou ressonância magnética cardíaca, foi realizada para investigar mais profundamente a gravidade da IA ou o mecanismo de regurgitação.^27 , 28^ Além disso, a falta de um método padrão-ouro impossibilitou uma avaliação mais precisa do valor da IVT. Ainda, os intervalos de valores da IVT obtidos fizeram com que o cálculo de um ponto de corte válido se tornasse impreciso. Assim, o baixo número de pacientes incluídos impediu uma validação transversal da IVT mensurada, tornando difícil chegar a conclusões sólidas. Por fim, não foi realizado acompanhamento clínico da amostra, o que impossibilita saber se o valor da IVT tem implicações clínicas ou prognósticas.

## Conclusões

A IVT da IA é um parâmetro ultrassonográfico facilmente obtido e reprodutível que parece estar associado com a gravidade da IA. Mais estudos são necessários para avaliar se este parâmetro é capaz de fornecer informações diagnósticas e prognósticas adicionais para pacientes com IA e se ele é útil em outras situações clínicas, como em casos de fibrilação atrial e em indivíduos com prótese valvar.
