# Supplementary material for: Integral Velocidade-Tempo da Insuficiência Aórtica: Um Novo Marcador Ecocardiográfico na Avaliação da Gravidade da Insuficiência Aórtica
Source: Arq Bras Cardiol. 2020 Aug 19;115(2):253–60. [Article in Portuguese] doi: 10.36660/abc.20190243 (PMC8384281; doi:10.36660/abc.20190243)
Supplement: Translation

# Translation: Velocity-Time Integral of Aortic Regurgitation: A Novel Echocardiographic Marker in the Evaluation of Aortic Regurgitation Severity

**Authors:** José Abellán-Huerta, Juan Carlos Bonaque-González, Ramón Rubio-Patón, José García-Gómez, Santiago Egea-Beneyto, Federico Soria-Arcos, Luciano Consuegra-Sánchez, Rosa María Soto-Ruiz, José Luis Ramos-Martín, Juan Antonio Castillo-Moreno

**Affiliations:** 1 Spain Departamento de Cardiologia, Hospital General Universitario Santa Lucía,Cartagena, Spain

**Keywords:** Heart Failure, Aortic Valve Insufficiency/diagnosis,imaging, Echocardiography, Doppler/methods

## Abstract

**Background:**

Echocardiography is essential for the diagnosis and quantification of aortic regurgitation (AR). Velocity-time integral (VTI) of AR flow could be related to AR severity.

**Objective:**

This study aims to assess whether VTI is an echocardiographic marker of AR severity.

**Methods:**

We included all patients with moderate or severe native AR and sinus rhythm who visited our imaging laboratory from January to October 2016. All individuals underwent a complete echocardiogram with AR VTI measurement. The association between VTI and AR severity was analyzed by logistic regression and multivariate regression models. A p-value<0,05 was considered statistically significant.

**Results:**

Among the 62 patients included (68.5±14.9 years old; 64.5%: moderate AR; 35.5%: severe AR), VTI was higher in individuals with moderate AR compared to those with severe AR (2.2±0.5 m vs. 1.9±0.5 m, p=0.01). Patients with severe AR presented greater values of left ventricular end-diastolic diameter (LVEDD) (56.1±7.1 mm vs. 47.3±9.6 mm, p=0.001), left ventricular end-diastolic volume (LVEDV) (171±36.5 mL vs. 106±46.6 mL, p<0.001), effective regurgitant orifice (0.44±0.1 cm^2^vs. 0.18±0.1 cm^2^, p=0.002), and regurgitant volume (71.3±25.7 mL vs. 42.5±10.9 mL, p=0.05), as well as lower left ventricular ejection fraction (LVEF) (54.1±11.2% vs. 63.2±13.3%, p=0.012). The VTI proved to be a marker of AR severity, irrespective of LVEDD, LVEDV, and LVEF (odds ratio 0.160, p=0.032) and of heart rate and diastolic blood pressure (DBP) (odds ratio 0.232, p=0.044).

**Conclusions:**

The VTI of AR flow was inversely associated with AR severity regardless of left ventricular diameter and volume, heart rate, DBP, and LVEF. VTI could be a marker of AR severity in patients with native AR and sinus rhythm. (Arq Bras Cardiol. 2020; [online].ahead print, PP.0-0)

## Introduction

Aortic regurgitation (AR) is one of the most common valvular disorders in the developed world.^1^ Typical management of the condition involves a combination of clinical signs and symptoms and data collection through complementary testing. Echocardiography is a key tool for the diagnosis and quantification of AR,^2^ and its proper interpretation requires an approach integrating qualitative, semiquantitative, and quantitative measures and parameters.^3 , 4^ However, these parameters are not exempt from limitations.^3^

Velocity-time integral (VTI) is defined as the area measured below the Doppler velocity curve at any given point. In the case of AR, its value corresponds to the diastolic pressure gradient between the aorta and the left ventricle (LV).^5^ In patients with AR, the VTI is multiplied by the aortic effective regurgitant orifice (ERO) to calculate the regurgitant volume (RV) (RV=EROxVTI).^2 , 6 , 7^ This parameter has demonstrated its effectiveness in determining AR severity, even though the ERO value is calculated using the proximal isovelocity surface area (PISA) method, which is known to have inherent limitations in patients with AR.^3 , 8 , 9^ Additionally, taking into account the aforementioned equation, patients with severe AR typically have larger RV^2^ and ERO values,^7 , 10^ but there is no evidence of the behavior of VTI in relation to AR severity.

Furthermore, patients with severe AR usually present increased end-diastolic pressure in the LV^11^ as well as reduced diastolic blood pressure (DBP).^12 , 13^ These pressure changes can pathophysiologically decrease VTI by reducing the pressure gradient between the aorta and LV. This study aimed to determine whether VTI is an echocardiographic marker of AR severity.

## Methods

### Study design and population

This retrospective cross-sectional observational study was performed over ten months (from January to October 2016). All patients with AR who visited our cardiac imaging laboratory during this period were eligible to participate. Patients had to exhibit moderate to severe AR in a native (non-prosthetic) valve as well as sign an informed consent form to be included in the study. We excluded patients with atrial fibrillation or evidence of any type of arrhythmia, multiple or eccentric jets of AR. The study complied with the declaration of Helsinki and was approved by the ethics committee of our local research panel.

### Baseline Characteristics of the Population

We gathered the following demographic and clinical information from all study participants: age, gender, history of arterial hypertension, dyslipidemia, diabetes mellitus, and smoking habits. Any type of antihypertensive, hypolipidemic, or antiarrhythmic drugs that the subjects were taking at the time of their inclusion in the study was also recorded. During the echocardiogram, the height and weight of each patient were collected, and three arterial blood pressure measurements were taken after 5 minutes of rest, using an M6 Comfort HEM-7221-E8 (Omron Healthcare, Kyoto, Japan) blood pressure monitor – validated through Dabl®Educational Trust and British Hypertension Society protocols –, following the European Society of Hypertension/European Society of Cardiology (ESH/ESC) recommendations.^14^ The final arterial blood pressure was the average of the second and third values. Heart rate (HR) was determined at the moment of the measurement of the VTI of AR. All patients also had a blood test performed immediately after collection to determine the plasma creatinine level and calculate the glomerular filtration rate using the CKD-EPI (Chronic Kidney Disease – Epidemiology Collaboration) formula.^15^ The blood test analyzer used was a PE Chemistry (Roche Diagnostics, Manheim, Germany).

### Echocardiographic Variables

Echocardiograms were performed on all subjects with an Acuson Siemens SC2000 ultrasound system. We used the Simpson’s biplane method to obtain standard measurements, images, and clips, including left ventricular end-diastolic diameter (LVEDD), left ventricular end-systolic diameter (LVESD), and left ventricular ejection fraction (LVEF), in accordance with recommendations from the American Society of Echocardiography.^16^ Both thickness and diameter were determined in M-mode with proper alignment whenever possible; otherwise, measurements were made in 2D. The measurement of the VTI of AR flow was taken using continuous Doppler readings from the view with the best alignment with the regurgitant jet, mainly the apical 5-chamber view ( Figure 1 ) or the parasternal long-axis view in cases of vertical regurgitant jet. Given that HR behaves as a temporal determinant of aortic VTI, the VTI index (VTIi) was calculated in addition to the absolute VTI value by dividing VTI by HR (VTIi=VTI/HR). The morphology of aortic valves was examined from the parasternal short-axis view. The systolic diameters of the right and left ventricular outflow tract were also measured. Pressure half-time (PHT) was calculated using the apical 5-chamber view. The vena contracta (VC) was estimated with color Doppler in two orthogonal planes, according to the recommendations.^16^ ERO was calculated based on the PISA method.^10 , 17^ To that end, images of the regurgitant flow were obtained using the best possible view for the alignment of the convergent flow. When zoomed in at this view, the color Doppler scale was optimized until the isovelocity hemisphere could be adequately differentiated. The PISA radius was measured between the first aliasing circumference relative to the center of the hemisphere in protodiastole, at the exact moment that the regurgitant flow reaches its maximum velocity. The RV was defined as the ERO x VTI product. Additionally, whenever possible, the RV was also determined quantitatively by estimating the aortic and pulmonary systolic volume.^18^ The flow reversal in the thoracic aorta was established using Pulse Doppler in the proximal end of the descending aorta through the suprasternal view. Holodiastolic flow with end-diastolic velocity >20 cm/s was considered a positive flow reversal. Finally, following a comprehensive and integrative analysis of the different structural, qualitative Doppler and the semiquantitative parameters obtained and taking into account the latest recommendations,^3 , 6^ two experienced echocardiographers separately quantified the AR. A third experienced echocardiographer assessed and conclusively quantified the AR in case of discordance between the two first cardiologists.

**Figure 1:** – *Measurement of the velocity-time integral of aortic regurgitation flow from the apical 5-chamber view. MnPG: mean pressure gradient; PG: maximum pressure gradient; Vmax: maximum aortic regurgitant flow velocity; VTI: velocity-time integral of aortic regurgitation.*

### Statistical Analysis

We tested all variables for normal distribution using the Kolmogorov-Smirnov test. Continuous variables with normal distribution were expressed as mean±standard deviation (SD), and those with skewed distribution as median [interquartile range (IQR)]. Categorical variables were expressed as percentages. Correlations were studied through Spearman’s or Pearson’s method, as appropriate. Inter-rater variability for AR severity quantification was determined by the intraclass correlation coefficient and Bland-Altman plots.^19^ Reliability analyses using kappa statistics (κ) defined the consistency between the two echocardiographers regarding AR severity (moderate or severe). Baseline differences between moderate or severe AR patients were assessed by unpaired Student’s *t* -test or Mann–Whitney U test for continuous variables and the χ^2^test for categorical variables. Logistic regression analysis evaluated the association between each baseline variable and severe AR. Multivariate logistic regression models determined the variables independently associated with severe AR. The variables included were those with p<0.05 in the univariate analysis, excluding RV, ERO, and VC, as they were not available for all patients and could cause overfitting. Model performance for predicting severe AR was evaluated by calibration (Hosmer-Lemeshow statistic) and discrimination (C-index) measures, both internally validated using the bootstrap resampling technique. The association between the VTI of AR and its severity was explored through multivariate analysis regardless of HR and DBP. We evaluated the relationship between VTIi and AR severity with a new logistic regression analysis. Confidence intervals (95%CI) were provided when appropriate. All probability values were 2-sided, and p-value<0.05 was statistically significant. Statistical analysis was performed using the SPSS software, v.18.0 (SPSS Inc., Chicago, Illinois).

## Results

The original sample consisted of 65 patients with moderate or severe native AR in sinus rhythm. Proper Doppler alignment of the regurgitant jet could not be obtained for three patients, who showed very eccentric jets, and were thus excluded. Out of the remaining 62 participants, 40 (64.5%) presented moderate AR, and 22 (35.5%) had severe AR. Acute AR was diagnosed in 4 patients (6.5% of the sample). The consistency among the quantification determined by the two echocardiographers was κ=0.83. All patients included were Caucasian. Table 1 presents the baseline characteristics of the sample.

**Table 1:** – Baseline characteristics

Characteristic	Total (n=62)	Moderate AR (n=40)	Severe AR (n=22)	p-value
**Age (years)**	68.5±14.9	68.6±14.2	66.1±15.5	0.299
**Male**	33 (53.2)	20 (50)	13 (59.1)	0.492
**BMI (kg/m** ^**2**^ **)**	27.5±4.7	26.5±4	29.4±5.9	0.340
**SBP (mmHg)**	135.6±17.8	133.6±16.7	139.4±19.8	0.213
**DBP (mmHg)**	62.2±15.5	63.2±12.7	59.8±19.8	0.373
**Heart rate**	66.8±11.3	65.8±10.6	68.5±12.4	0.382
**Arterial hypertension**	45 (72.6)	29 (72.5)	16 (72.7)	0.985
**Diabetes mellitus**	11 (17.7)	8 (20)	3 (16.6)	0.530
**Dyslipidemia**	30 (48.4)	20 (50)	10 (45.5)	0.732
**Active smokers**	10 (16.1)	6 (15)	4 (18.2)	0.744
**eGFR (mL/kg/1.73 m** ^**2**^ **)**	77.3 [40.3]	86.6 [42.3]	72.9 [34.6]	0.408
**Hemoglobin**	13.2±1.8	13.3±1.8	13.2±2	0.893
**Beta-blockers**	29 (46.8)	17 (42.5)	12 (54.5)	0.363
**ACE inhibitors**	19 (30.6)	11 (27.5)	8 (36.4)	0.469
**ARA**	16 (25.8)	13 (32.5)	3 (13.6)	0.104
**DHP CCB**	2 (3.2)	2 (5)	0 (0)	0.286
**Non-DHP CCB**	10 (16.1)	4 (10)	6 (27.3)	0.145
**Amiodarone**	2 (3.2)	1 (2.5)	1 (4.5)	1
**Diuretics**	31 (50)	17 (42.5)	14 (63.6)	0.111
**Statins**	26 (41.9)	19 (47.5)	7 (31.8)	0.231
**Previous hospital admission for HF**	16 (25.8)	9 (22.5)	7 (31.8)	0.422

*ACE: angiotensin-converting enzyme; AR: aortic regurgitation; ARA: angiotensin receptor antagonists; BMI: body mass index; CCB: calcium channel blockers; DBP: diastolic blood pressure; DHP: dihydropyridine; eGFR: estimated glomerular filtration rate; HF: heart failure; SBP: systolic blood pressure. Continuous variables with normal distribution are expressed as mean±standard deviation, those with skewed distribution as median [interquartile range], and categorical variables as n (percentage).*

As shown in Table 2 , the VTI of the aortic regurgitant flow was higher in patients with moderate AR than in those with severe AR. The VTI range was 2.05 m [1.53–3.58 m] in the moderate AR group and 1.88 m [0.96–2.84 m] in the severe AR group. We found a significant and inverse correlation between VTI and HR [Pearson’s correlation coefficient (r_p_)=-0.408, p=0.001]. Patients with severe AR presented lower LVEF, higher LVEDD and LVESD, as well as a larger ERO, RV, and VC. However, the proper measurement of these parameters was only possible in 62.9% of the sample for ERO, 67.7% for RV, and 72.6% for VC. We underline that we identified no statistically significant association between AR severity and PHT, even though we detected a trend for it.

**Table 2:** – Values of echocardiographic parameters

Parameter	Total (n=62)	Moderate AR (n=40)	Severe AR (n=22)	p-value
**AR VTI (m)**	2.1±0.5	2.2±0.5	1.9±0.5	0.010
**AR VTIi (VTI/heart rate)**	0.033±0.012	0.036±0.013	0.028±0.01	0.024
**Aortic PHT (ms)**	397.3±110.1	434.2±127	367.5±86.2	0.062
**Vena contracta (mm)**	6±1.5	5.5±1.5	7.1±1.2	0.035
**ERO (cm** ^**2**^ **)**	0.31±0.2	0.18±0.1	0.44±0.1	0.002
**Regurgitant volume (mL)**	56.9±24	42.5±10.9	71.3±25.7	0.05
**Thoracic aorta flow reversal**	33 (53.2)	12 (30.8)	21 (95.5)	<0.001
**IV septum thickness (mm)**	13.1±3.6	12.5±3.5	13.8±3.5	0.460
**Posterior wall thickness (mm)**	10.6±2.8	10.3±2.7	11±3.1	0.383
**LVEDD (mm)**	50.5±9	47.3±9.6	56.1±7.1	0.001
**LVESD (mm)**	31±11.4	26.9±12.3	38.4±8.1	<0.001
**LVEDV (mL)**	131.9±54.3	106±46.6	171±36.5	<0.001
**LVESV (mL)**	53.6±36.1	39.9±32.2	78.7±27.5	<0.001
**LVEF (%)**	59.7±13.2	63.2±13.3	54.1±11.2	0.012
**AR peak velocity (m/s)**	4.2±0.51	4.3±0.5	4.1±0.52	0.344
**Aortic systolic peak velocity (m/s)**	2.7±1.2	2.8±1.4	2.7±0.9	0.791
**Bicuspid aortic valve**	5 (8.1)	3 (4.8)	2 (3.2)	0.826
**Elevated LV filling pressure**	26 (41.9)	16 (42.1)	10 (50)	0.566
**Severe mitral regurgitation**	2 (3.2)	2 (5)	0 (0)	0.286
**Severe mitral stenosis**	1 (1.6)	1 (2.5)	0 (0)	0.455
**Severe aortic stenosis**	8 (12.9)	6 (15)	2 (9.1)	0.507

*AR: aortic regurgitation; ERO: effective regurgitant orifice; IV: interventricular; LV: left ventricle; LVEF: left ventricular ejection fraction; LVEDD: left ventricular end-diastolic diameter; LVEDV: left ventricular end-diastolic volume; LVESD: left ventricular end-systolic diameter LVESV: left ventricular end-systolic volume; PHT: pressure half-time; VTI: velocity-time integral; VTIi; velocity-time integral index. Continuous variables with normal distribution are expressed as mean±standard deviation and categorical variables as n (percentage).*

In the bivariate analysis ( Table 3 ), VTI was inversely associated with AR severity. Besides, the classic severity variables related to the size and function of the left ventricle were associated with AR severity. In the multivariate analysis, the VTI value acted as a marker of AR severity regardless of LVEDD, left ventricular end-diastolic volume (LVEDV), and LVEF ( Table 4 ). LVESD and left ventricular end-systolic volume were excluded from the multivariate analysis due to collinearity with LVEDD (r_p_=0.905, p<0.001) and LVEDV (r_p_=0.871, p<0.001), respectively. We also excluded ERO, RV, and VC from the multivariate analysis as they could not be obtained for all patients due to poor ultrasound window or difficulty in measuring. This model showed greater discrimination (Statistic C=0.837, 95%CI 0.728–0.947) and an accurate calibration (Hosmer-Lemeshow χ^2^=2.30, p=0.970).

**Table 3:** – Bivariate logistic regression model (dependent variable: severe aortic regurgitation)

	Odds ratio	95%CI	p-value
AR VTI	0.198	0.053–0.748	0.017
AR VTIi	<0.001	<0.001–0.005	0.033
LVEF	0.941	0.895–0.989	0.017
LVEDD	1.144	1.047–1.249	0.003
LVESD	1.119	1.044–1.199	0.001
LVEDV	1.032	1.015–1.049	<0.001
LVESV	1.034	1.013–1.057	0.002

*95%CI: 95% confidence interval; AR VTI: velocity-time integral of aortic regurgitation; AR VTIi: velocity-time integral index of aortic regurgitation; LVEDD: left ventricular end-diastolic diameter; LVEDV: left ventricular end-diastolic volume; LVEF: left ventricular ejection fraction; LVESD: left ventricular end-systolic diameter; LVESV: left ventricular end-systolic volume.*

**Table 4:** – Multivariate logistic regression model (dependent variable: severe aortic regurgitation)

	Odds ratio	95%CI	p-value
AR VTI	0.160	0.030–0.856	0.032
LVEF	1.005	0.933–1.082	0.895
LVEDD	1.049	0.934–1.178	0.419
LVEDV	1.030	1.009–1.052	0.005

	**Odds ratio**	**95%CI**	**p-value**

AR VTIi	<0.001	<0.001–<0.001	0.019
LVEF	1.007	0.932–1.089	0.859
LVEDD	1.063	0.939–1.204	0.333
LVEDV	1.032	1.010–1.055	0.005

*95%CI: 95% confidence interval; AR VTI: velocity-time integral of aortic regurgitation; AR VTIi: velocity-time integral index of aortic regurgitation; LVEDD: left ventricular end-diastolic diameter; LVEDV: left ventricular end-diastolic volume; LVEF: left ventricular ejection fraction.*

On the other hand, since HR and DBP could pathophysiologically influence the VTI measurement (DBP as a determinant of velocity, and HR of time), the association between VTI and AR severity was assessed adjusting for HR and DBP. VTI was also inversely related to AR severity, irrespective of these factors (OR 0.232, 95%CI 0.056–0.961, p=0.044). Finally, the VTIi of AR also showed an inverse association with AR severity ( Table 3 ) and acted as a marker of AR severity, regardless of LVEDD, LVEDV, and LVEF in the multivariate analysis ( Table 4 ). Additionally, this variable was also related to AR severity, irrespective of DBP (OR<0.001, 95%CI <0.001–0.001, p=0.029).

## Discussion

This study suggests that the VTI of AR can be used as a marker of severity in patients with significant AR, considering that estimating severity through echocardiography is a difficult process involving the integration of several different tests and parameters.^2 - 4 , 20^

Effectively, ERO by the PISA method works as a parameter for the stratification of AR severity,^3 , 7^ and an indirect relationship can be found between ERO and VTI (ERO=RV/VTI).^3 , 10^ More severe AR presents a larger ERO and RV, but the behavior of VTI is unknown. In this study, the VTI of the aortic regurgitant flow was inversely associated with AR severity. The scientific evidence available corroborating this relationship is scarce. Zarauza et al.^21^ published a study that assessed the value of VTI of AR, amongst other parameters, in a sample of 43 patients with moderate to severe AR.^21^ Their findings were similar to ours (severe AR VTI: 1.8±0.7 m vs. 1.9±0.5 m; moderate AR VTI: 2.2±0.8 m vs. 2.2±0.5 m, respectively). However, in the study by Zarauza et al.,^21^ the differences between VTI in severe and moderate AR did not reach statistical relevance. The difference in sample size for patients with moderate AR (15 vs. 40) could explain the lack of a significant result. To the best of our knowledge, no other study has assessed the value of VTI as an indicator of AR severity.

A remarkable aspect of the present study is the direct association between AR severity and end-diastolic and end-systolic diameters and volumes, in addition to the inverse relationship to LVEF. These findings are consistent with available scientific evidence, which supports the predictive role of left ventricular diameter and ventricular function as markers of advanced AR and negative prognoses.^11 , 22 - 24^ In our opinion, this aspect reflects an appropriate and rigorous methodology for measuring these parameters. This study found that the relationship between VTI and AR severity did not depend on echocardiographic variables, such as left ventricular diameters, volumes, or ejection fraction. This result could potentially support the use of VTI as an indicator in most echocardiographic scenarios involving AR and sinus rhythm.

Despite being echocardiographic methods recommended for determining the severity of significant AR,^3 , 25 , 26^ the calculations necessary to estimate VC, RV, and, as we previously mentioned, ERO obtained by PISA present several limitations.3,8,9,17 In fact, this study could not evaluate whether the VTI value was associated with severe AR, regardless of ERO, RV, or VC, as the percentage of patients from whom this data could be obtained was not enough to perform a valid multivariate analysis. In contrast, VTI could not be estimated in only 3 of the 65 patients of this study due to improper alignment of the AR jet. Thus, VTI has proven to be a reproducible parameter that can be easily obtained and examined in most patients and could provide valuable information for the stratification of AR severity.

We also emphasize that although PHT was obtained for all patients who had their VTI calculated, we found no significant differences between individuals with moderate and severe AR, which prevented the inclusion of this parameter in the multivariate analysis. Therefore, we could not assess the additional value of VTI with respect to PHT. Current clinical guidelines suggest that the usefulness of PHT is low in cases of chronic AR,^2 , 3^ and the sample of the present work consists mainly of chronic AR patients. The low rate of acute AR in the present study (6.5%) precluded a feasible statistical evaluation of the acute AR cohort. This issue could explain the lack of differences in the PHT values between the moderate and severe AR groups.

Our results also suggest that the association between lower VTI and severe AR does not seem to be significantly affected by hemodynamic variables, such as HR and DBP. If other studies supported this relationship behavior, the use of VTI could reach a wide variety of patients. However, we consider that the relationship between VTI and AR severity could not be significantly changed by these hemodynamic variables due to a lack of extreme values. We highlight that we found a tendency for lower DBP in patients with severe AR and that we excluded patients with atrial fibrillation. Thus, since HR is a temporal determinant for the VTI of AR, we also calculated the VTI indexed by HR to normalize the VTI value and further study its relationship to AR severity. Additionally, HR correlated significantly and inversely with AR VTI. The relationship between this new variable and AR severity was not only maintained but proved to be stronger and independent of LVEDD and LVEF (OR<0.001, p=0.031). In other studies, such as the one by Zarauza et al.,^21^ VTI was normalized using the diastolic length.^21^ However, there are few levels of consistency throughout the indexing of VTI in terms of HR. We believe that these findings support the pathophysiological hypothesis that a smaller VTI is associated with a more severe AR, regardless of the HR.

Our study presents several limitations. First, this is a single-center study that did not analyze patients with AR and atrial fibrillation or prosthetic valves, and thus the value of the aortic VTI in those subpopulations is unknown. Second, the VTI was obtained by Doppler imaging, and, therefore, it is subject to the limitations of this technique. Additionally, our analyses only included patients with moderate or severe AR in an attempt to avoid a potential underestimation in the VTI measurement of low-density mild regurgitant jets; thus, the usefulness of VTI in determining the severity of mild AR remains unclear. ERO, RV, and VC could not be obtained for all patients, partially due to the retrospective nature of the present paper, preventing the assessment of the VTI value with respect to these parameters in predicting severe AR in a multivariate analysis. No other exploration techniques, such as transesophageal echocardiogram, 3D ultrasound, or cardiac magnetic resonance, were performed to study the AR severity or the mechanism of regurgitation in depth.^27 , 28^ Moreover, the lack of a gold standard method precluded a more accurate assessment of the VTI value. Otherwise, the ranges of the VTI values obtained made inaccurate the calculation of a valid cut-off point. Thus, the small number of patients included prevented a cross-sectional validation of the VTI measured, making it difficult to reach solid conclusions. Lastly, we performed no clinical follow-up of the sample, making it impossible to know whether the VTI value has any prognostic or clinical implications.

## Conclusions

The VTI of AR is an easily obtainable and reproducible ultrasound parameter that seems to be associated with AR severity. Further studies are necessary to evaluate whether this parameter is capable of providing additional diagnostic and prognostic information for patients with AR, and whether it is useful in other clinical scenarios, such as atrial fibrillation and in individuals with prosthetic valves.
